# Determination of the Electrochemical Area of Screen-Printed Electrochemical Sensing Platforms

**DOI:** 10.3390/bios8020053

**Published:** 2018-06-08

**Authors:** Alejandro García-Miranda Ferrari, Christopher W. Foster, Peter J. Kelly, Dale A. C. Brownson, Craig E. Banks

**Affiliations:** 1 Faculty of Science and Engineering, Manchester Metropolitan University, Manchester M1 5GD, UK; alejandro.garcia-miranda-ferrari@stu.mmu.ac.uk (A.G.-M.F.); cwfoster90@gmail.com (C.W.F.); peter.kelly@mmu.ac.uk (P.J.K.); d.brownson@mmu.ac.uk (D.A.C.B.); 2 Manchester Fuel Cell Innovation Centre, Manchester Metropolitan University, Manchester M1 5GD, UK

**Keywords:** screen-printed electrodes, real electroactive area, cyclic voltammetry, chronocoulometry, Anson plot, Randles–Ševćik

## Abstract

Screen-printed electrochemical sensing platforms, due to their scales of economy and high reproducibility, can provide a useful approach to translate laboratory-based electrochemistry into the field. An important factor when utilising screen-printed electrodes (SPEs) is the determination of their real electrochemical surface area, which allows for the benchmarking of these SPEs and is an important parameter in quality control. In this paper, we consider the use of cyclic voltammetry and chronocoulometry to allow for the determination of the real electrochemical area of screen-printed electrochemical sensing platforms, highlighting to experimentalists the various parameters that need to be diligently considered and controlled in order to obtain useful measurements of the real electroactive area.

## 1. Introduction

Screen-printed electrochemical platforms form the basis of translating laboratory-based studies into industry for “in-the-field” experimentation. These platforms are fabricated on a large scale, resulting in low cost, yet highly reproducible sensors which can be used as either single-use (disposable) or can be readily modified via surface modification with enzymes, nanostructures, or even the bulk of the sensor can be adapted to allow bespoke applicability in a multitude of applications [1,2]. For example, Banks et al. [3,4] have shown that metal oxide (bismuth, antimony, and tin) bulk modified screen-printed electrodes can offer a suitable platform for the sensing of heavy metals. Additionally, screen-printed platforms have been utilized, both as-is (unmodified) and modified, within many sensing applications, for example, towards the detection of biomolecules (such as lactate [5] or L-cysteine [6]), gases (such as O_2_ [7] or CO [8]), and metals (such as Sb and Sn [9]); with particular success within the food industry, a key indication of their quality and versatility, with respect to capsaicin [10] and garlic [11] which have been successfully analysed with screen-printed electrodes (SPEs). An important parameter to consider when utilising electrochemical sensors is the real electroactive area, especially within fundamental calculations of electrochemical processes, as well as providing a methodology for their benchmarking with respect to the quality control of SPEs.

Jarzabek and Borkowska [12] have reported on determining the electrochemical area of gold polycrystalline electrodes using the mass transport and adsorption process of different oxide species (namely HClO_4_ and LiClO_4_). While Czervinski et al. [13] have provided a thorough overview of the various approaches to determine the electroactive area of noble metal electrodes, reporting that each method implemented to measure the electroactive area of an electrode is based upon very specific theory and assumptions, thus not providing a standardised method for the determination of these metal based electrodes.

The determination of the real electroactive surface area of SPEs has also been investigated using the redox probe *N*,*N*,*N*’,*N*’-tetramethylphenyl-diamine (TMPD) [14], through the popular methodologies of cyclic voltammetry (CV) and chronocoulometry (CC). Interestingly, the approach via chronocoulometry has been used to determine the real electrochemical area of nitrogen-doped graphene modified electrodes, with Ru(NH_3_)_6_Cl_3_^3+/2+^ used as the redox probe [15]. Other groups have also used CC to measure the area of gold nanostructures [16], CNT/NiO microfluidic electrode [17], and Ag@Pt nanorods [18] using ferrocyanide/ferricyanide redox probes. Additionally, alternative electrochemically irreversible probes such as NAD^+^ and ascorbic acid have been used to measure the surface area of commercial SPEs using CV [19]. Moreover, Terranova et al. have utilised dilute H_2_SO_4_ for determining the area of a MWCNT-PtNP modified electrode using a both CV and CC methods [20].

In the literature, in the above reported methodologies, such measurements are undertaken with little understanding or consideration for the redox probe utilised or experimental parameters and this same literature does not properly explain this to novices. Consequently, in this paper, we consider the case of the determination of the real electroactive area of SPEs, using both CV and CC, providing a robust guide for experimentalists.

## 2. Materials and Methods

All chemicals used were analytical grade and were used as received from Sigma-Aldrich (Irvine, UK) without any further purification. All solutions were prepared with deionised water of resistivity no less than 18.2 MΩ cm and were vigorously degassed prior to electrochemical measurements with high purity, oxygen free nitrogen. The tested solutions were: 1 mM *N*,*N*,*N*′,*N*′-tetramethyl-*p*-phenylenediamine (TMPD) in 0.1 M KCl, 1 mM Ru(NH_3_)_6_Cl_3_^3+/2+^ (RuHex) in 0.1 M KCl, 1 mM Dopamine in pH 7 phosphate buffer solution/0.1 M KCl (PBS), 1 mM *β*-Nicotinamide adenine dinucleotide (NADH) in pH 7 PBS/0.1 M KCl, 1 mM Capsaicin in 0.1 M HPO_4_, and 1 mM Ascorbic acid in pH 7 PBS/0.1 M KCl. The following diffusion coefficients were used in this work (cm^2^ s^−1^): 6.74 × 10^−6^ for dopamine [21], 7.40 × 10^−6^ for NADH [22], 9.1 × 10^−6^ for RuHex [23], 6.32 × 10^−6^ for TMPD [24], 7.03 × 10^−6^ for capsaicin, and 1.42 × 10^−6^ for ascorbic acid [25].

Electrochemical measurements were performed using an Autolab PGSTAT204 (Metrohm Autolab, Utrecht, The Netherlands) computer-controlled potentiostat. All measurements were conducted using a three-electrode system with a Pt wire counter electrode, a saturated calomel electrode (SCE) reference electrode, and screen-printed graphite working electrodes (SPEs) completing the circuit. The SPEs were fabricated in-house with appropriate stencil designs to achieve a 3.1 mm diameter working electrode, using a carbon-graphite ink (Product Ink: C2000802P2; Gwent Electronic Materials Ltd., Pontypool, UK) printed using a DEK 248 screen printer machine (DEK, Weymouth, UK) onto a polyester (Autostat, Milan, Italy, 250 micron thickness) flexible film. This layer was cured in a fan oven at 60 °C for 30 min and finally, a dielectric paste (Product Code: D2070423D5; Gwent Electronic Materials Ltd., Pontypool, UK) was then printed onto the polyester substrate to cover the connections. After a second curing process at 60 °C for 30 min, the SPEs are ready to be used. The in-house fabricated SPEs have been previously reported and characterized [26,27]. The electrode’s roughness was calculated using a Profilm3D white light interferometry (WLI) (Filmetric, San Diego, CA, USA).

The electrode areas calculated using the Randles–Ševćik equation and cyclic voltammetry were undertaken with 10 different scan rates (5, 10, 15, 25, 50, 75, 100, 150, 250, and 500 mV s^−1^). For the area calculated using the Anson equation and chronocoulometric experiments, CC, two potentials were applied. The first potential was applied at a low voltage where no electrochemical Faradaic reaction occurred, and the second potential was applied in order detect the corresponding Faradaic process; total charge passed versus time was recorded for 6 s.

## 3. Results and Discussion

The electrode area of screen-printed electrodes (SPEs) can be physically/visually determined with techniques such as scanning electron microscopy. In this case, the geometrical area, *A_geo_*, is determined through its physical dimensions, but there is no resemblance to the true electroactive area and there is no way of knowing which parts of the electrode surface are electrochemically active or indeed inactive. The most appropriate way is to use an interfacial technique such as electrochemistry; it is this approach that we consider herein.

### 3.1. Determining the Electroactive Area Using Cyclic Voltammetry

Firstly, we must consider the respective Randles–Ševćik equations (at non-standard conditions) [28,29,30,31] for reversible, quasi-reversible, and irreversible electrochemical processes:

(1) $$I_{p,f}^{rev}=\pm 0.446\;nFA_{real}C\;\sqrt{\;\frac{nFD\nu }{RT}}$$

(2) $$I_{p,f}^{quasi\;}=\;\pm 0.436\;nFA_{real}C\;\sqrt{\;\frac{nFD\nu }{RT}\;}$$

(3) $$I_{p,f\;}^{irrev\;}\;=\;\pm 0.496\;\sqrt{\alpha n'}\;nFA_{real}C\;\sqrt{\frac{nFD\nu }{RT}\;}$$

where in all cases, *n* is the number of electrons in the electrochemical reaction, *I_p,f_* is the voltammetric current (analytical signal) using the forward peak of the electrochemical process, *F* is the Faraday constant (C mol^−1^), *v* is the applied voltammetric scan rate (V s^−1^), *R* is the universal gas constant, *T* is the temperature in Kelvin, $A_{real}$ is the electroactive area of the electrode (cm^2^) and *D* is the diffusion coefficient (cm^2^ s^−1^), *α* is the transfer coefficient (usually assumed to be close to 0.5), and *n’* is the number of electrons transferred before the rate determining step. Equations (1)–(3) can be used to determine the electroactive area (*A_real_*) through a simple cyclic voltammetry experiment. In this approach, typically, a reliable redox probe within an aqueous electrolyte is used to determine a plot of the *forward* peak current, *I_p,f_*, as a function of applied voltammetric scan rate (*v*^1/2^). This is since the Randles–Ševćik equation is derived from assuming that the concentration of the electroactive species (in the bulk solution) is the same as that at the electrode surface, due to the development of the diffusion layer [32]. 

There are important factors to consider when utilising the aforementioned Randles–Ševćik (R–S) equations:

(1) Which equation should be used for each redox probe utilised? i.e., which equation from (1)–(3) is the most suitable to use? Analysis of the peak-to-peak separation (*ΔEp*) of the recorded voltammogram is useful, where in the reversible limit the *ΔEp* is ~57mV and is independent of scan rate. In the case of quasi- and irreversible conditions, the *ΔEp* is larger and is dependent upon the voltage scan rate. The wave-shape of the forward peak allows one to determine between reversible and irreversible conditions; a full analysis is given in reference [32].
(2) The R–S equations should only be used for the forward scan [32], this is due to the fact that on the forward wave, the product is electrochemically produced and diffusion occurs, giving, as a result, a concentration of zero product within the bulk solution compared to that at the electrode surface. Consequently, on the return scan, returning the electrochemically formed product back to its starting material, a decrease in the concentration of the product has occurred, resulting in a less intense backward peak than the forward one. The Randles–Ševćik equations are only an approximation, and therefore do not represent an exact value, unlike, as for example, the case of chronocoulometry.
(3) The Randles–Ševćik equations are more suitable for macroelectrodes, therefore, which size of electrode can be utilized to satisfy the Randles–Ševćik equation? i.e., how big does the electrode need to be in order to give rise to the mass transport dominated by planar diffusion? Compton has undertaken experiments inferring that working electrodes of no less than 4 mm radius should be employed for investigations in aqueous solutions [33]. Their work demonstrates that for a simple electron transfer process, the *ΔEp* is reduced from 60.6 mV using a radius of 0.5 mm to 57.5 mV in the case of a radius of 4 mm and larger; the quantitative change is due to the geometric electrode size increasing such radial diffusion [33].
(4) One must consider, is the electrode relatively flat and non-porous? In order for Equations (1)–(3) to hold, this should be the case. In the case of a SPE, the electrode is heterogeneous, comprising a range of different carbons (graphite, carbon black) and binder(s). It should be noted that the surface roughness of a SPE is typically 0.078 µm (see Figure S1). Over the timescale of the voltammetric experiment, as determined by Compton [32,34], the diffusion layer is larger than the SPE micro-features such that the electrode kinetics are heterogeneous and dominated by the faster electrode material, i.e., the edge plane features of the graphite(s)/carbon black(s). In this case, Equations (1)–(3) hold; see references [32,34] for the categorisation of electrochemically heterogeneous surfaces that may be encountered.
(5) The potential window is not reversed too early, and the analysis of the forward peak is used on the first scan [32].
(6) The scan rate is not too fast to make the cyclic voltammetric response become non-reversible. This is since the Randles–Ševćik equations are derived from assuming the concentration of the electroactive species in the bulk is the same as that at the surface of the electrode, which, as highlighted above, is due to a diffusion layer developing [32].
(7) In the case of determining the electrode area, a reliable diffusion coefficient (D) value needs to be utilized. A useful approach is the Wilke–Chang [35] equation to determine the diffusion coefficient:
  (4) $$D=\;7.4\;\times \;10^{-8}\;\frac{T\sqrt{xMs}}{\eta V^{0.6}}$$
  where *x* is the association parameter to define the effective molecular weight of the solvent with respect to the diffusion process (where *x* = 2.6 for water and *x* = 1 for non-associated solvents [35]), *Ms* is the molecular weight of the solvent (g mol^−1^), *η* is the viscosity of the solution (g cm^−1^ s^−1^), and *V* is the molar volume of solute at normal boiling point (cm^3^ g^−1^ mol^−1^). This equation predicts the *D* value with an exponential error of ±13%. As highlighted by Sitaraman et al. [36], finding the association parameter (*x*) becomes an issue for unknown systems, therefore the following correction has been proposed:
  (5) $$D=5.4\;\times \;10^{-8}(\frac{TLs^{\frac{1}{3}}\sqrt{xMs}}{\eta V^{0.6}\sqrt{Vm}Ls^{0.3}}{)}^{0.93}$$
  where *Ls* is the latent heat of vaporization of solute at normal boiling point (cal g^−1^). This methodology still has an error of ±13%, but is simpler when used by experimentalists. Clearly, temperature is critical in determining the electrochemical area. Changes and fluctuations in temperature will affect the information obtained from Equations (1)–(5). Consequently, the temperature at the time of measuring the electrochemical area should be measured and factored into these equations.
(8) The Randles–Ševćik equations are useful for single electron transfer processes that feature a 1:1 reaction stoichiometry, inversely however, for example, the reduction of protons to hydrogen (hydrogen evolution reaction, HER) has a 2:1 stoichiometry and experimental results deviate from theory [37]. The diffusion coefficients used here are either from the academic literature or deduced using Equation (5).

**Figure 1:**
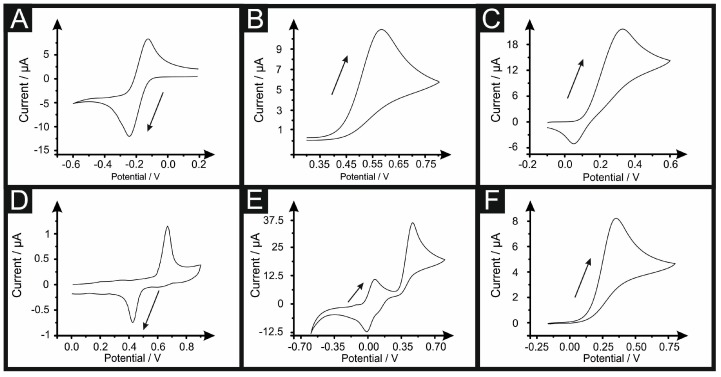
Cyclic voltammetry of 1 mM RuHex (**A**); 1 mM NADH (**B**); 1 mM dopamine (**C**); 1 mM capsaicin (**D**); 1 mM TMPD (**E**) and 1 mM ascorbic acid (**F**) at 0.05 V s^−1^ (vs. SCE) and 19.6 °C. The arrow indicates the forward peak selected to calculate the *A_geo_* with Equations (2) and (3).

Returning to the case of determining the electrode area, electrochemical experiments were performed using redox probes with a range of voltammetric scan rates utilised (see experimental section). The appropriate Randles–Ševćik equations were utilised to determine the electrode area (A*_real_*). These results are depicted in Figure 1, and their calculated *A_real_* and its percentage compared to the *A_geo_* (%*Real* = (*A_real_/A_geo_*) × 100) is reported in Table 1. (Note: in case of capsaicin, the second electrochemical process of the reaction was utilised and in case of the TMPD, the first oxidation peak was chosen). 

**Figure 2:**
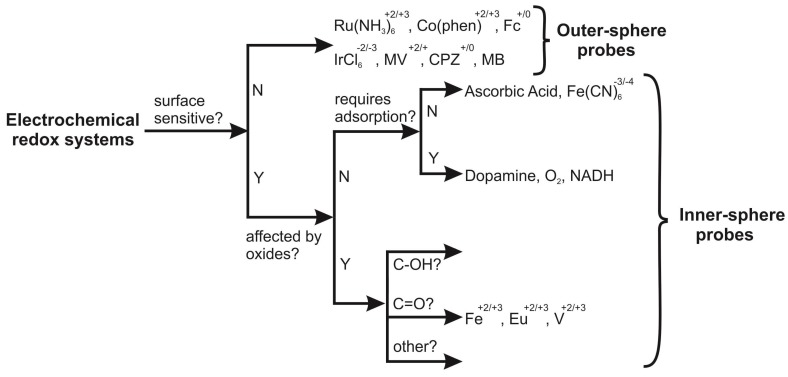
Classification of redox systems by Mcreery et al. [39] according to their kinetic sensitivity to particular surface modifications upon carbon electrodes. The figure has been adapted by the authors of this paper to clearly show outer- and inner-sphere probes. Fc: ferrocene, MV: methyl viologen, CPZ: chlorpromazine, and MB: methylene blue.

It is readily evident that a range of %*Real* values are obtained, alternating from 75 to 123%. Using SPEs, one can realise that this value will likely deviate from 100% due to the binder holding the graphite and carbon black components together comprising the electrode surface. So what value of %*Real* is the one to be utilised? One needs to not only consider the limitations of the Randles–Ševćik equations, as identified above, and the correct experimental criteria, but the redox probe used is also of importance. Figure 2 shows an elegant flow chart depicting the electrochemical processes of redox complexes, classifying each redox probe as either inner- or outer-sphere probes depending on how the electron transfer processes occur on the surface of the electrode. The Randles–Ševćik equations are only an approximation and this also strictly applies for diffusional control redox processes [32]. Redox process (see Figure 2) are classed into outer-sphere where electron transfer is fast where the redox probes comes close to the electrode surface for electrons to tunnel/hop across a monolayer of solvent but do not directly interact with the electrode surface. Such redox probes are influenced by the electronic structure of the electrode surface only. On the other hand, inner-sphere probes are influenced not only by the electronic structure but also the electrode surface (either reactant and/or product), i.e., surface functional groups (adsorption sites)/surface chemistry [38]. Thus, in the redox probes utilized, RuHex is a near ideal outer-sphere redox couple which does not show any changes in its electron transfer rates due to varying surface chemistry and is only dependent upon the electrode’s electronic structure (Density of States (DOS) and the Fermi level), thus allowing its use to give rise to the closest estimations of the real electrode area (*A_real_*).

### 3.2. Determining the Electroactive Area Using Chronocoulometry

**Figure 3:**
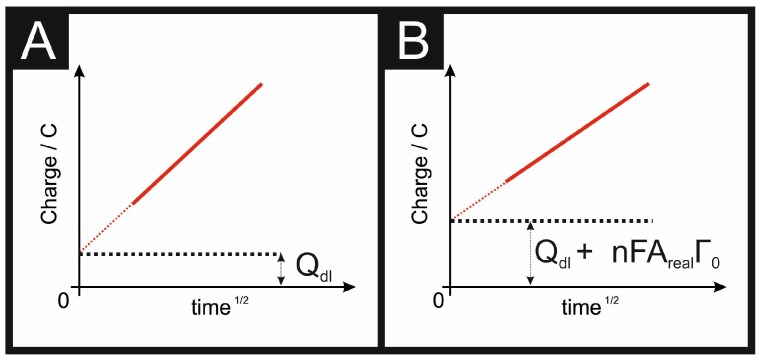
Representation of CC (charge vs. time^1/2^) for a non-adsorbed outer-sphere probe (**A**) and for an adsorbed inner-sphere probe (**B**). Note that, in (**A**), the intercept is not truly zero due to the contribution of $Q_{dl}$ (see Equations (6) and (7)).

Chronocoulometry (CC) is a classical electrochemical technique that has been overlooked in recent years. CC was developed by Anson [40] (see Equations (6) and (7)), and involves the measurement of charge vs. time response from an applied potential step waveform. The shape of the resulting chronocoulogram, is summarised within Figure 3. CC is a useful technique in electrochemistry allowing one to readily determine the real electrochemical active electrode area, as well as their respective diffusion coefficients, the time-window of an electrochemical cell, adsorption of electroactive species, and rate constants for chemical reactions coupled to electron transfer reactions; this, in summary, is a very useful electrochemical approach.

The Anson equation for diffusion-only processes (see Equation (6)) defines the charge–time dependence for linear diffusion control, using a CC method, which is the measure of charge (Coulombs) as a function of time, and can be applied to the measurement of the real electroactive area of electrodes [14]. In the case of adsorption, for electrochemical processes (such as the case of inner-sphere probes, see Equation (7) where the reactants are adsorbed, the charge is due to the electrolysis of the adsorbed species which can be distinguished from the charge that is occurring from the electrolysis of the solution-based species. The adsorbed materials upon the electrode surface are electrolyzed immediately upon application of the potential step, while the solution-based species obviously have to diffuse to the electrode surface to react. In this case, the total charge is the contribution of the double layer, the electrolysis of the adsorbed species, and the electrolysis of the solution.

Anson equations:

*for diffusion processes*

(6) $$Q=\;\frac{2nFA_{real}C\sqrt{Dt}}{\sqrt{\pi }}\;+Q_{dl\;}$$

*for adsorption processes*

(7) $$Q=\;\frac{2nFA_{real}C\sqrt{Dt}}{\sqrt{\pi }}+nFA_{real}\Gamma_{0}+Q_{dl}$$

where *Q* is the charge, *n* is the number of electrons in the electrochemical reaction, *A_real_* is the electroactive area, *F* is Faraday’s constant, *C* is the concentration, *D* is the diffusion coefficient, $Q_{dl}$ is the double layer capacitance, and $\Gamma_{0}$ is the coverage of adsorbed reactant (mol cm^−2^). The electrolysis of the diffusion species shows a dependence upon ${\mathrm{time}}^{1/2}$. Since the adsorbed materials are electrolyzed instantaneously, the charge is not time-dependant and the charging of the double layer is instantaneous and independent of time. Thus, as shown in Figure 3, the difference in the charge due to the $Q_{dl}+\;nFA_{real}\Gamma_{0}$ is readily evident but the gradient is the same, due to the reaction cited above. Therefore, CC is most accurate when considering near-ideal outer-sphere probes or highly adsorbed analytes.

**Figure 4:**
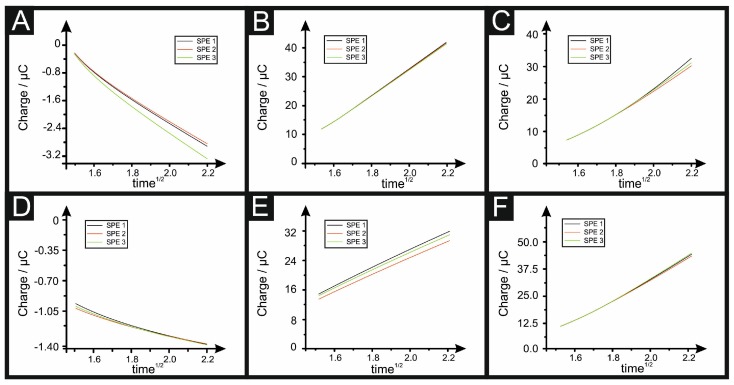
Anson plots resulting from using the following redox probes: 1 mM RuHex (**A**); 1 mM NADH (**B**); 1 mM dopamine (**C**); 1 mM capsaicin (**D**); 1 mM TMPD (**E**) and 1 mM ascorbic acid; (**F**) at 19.6 °C. The results of three different SPEs are presented.

CC was described by Anson to determine quantities of adsorbed reactants, as such, this equation cannot be reliably used to determine $A_{real}$, due to the contributions of $“nFA_{real}\Gamma_{0}”$ in the aforementioned equations (i.e., having two different terms in the equations representing the area). A range of redox probes were used to calculate the electroactive area of SPEs using the Anson plot (Equations (6) and (7)), applying a first potential pulse to reduce all of the analyte and a second pulse to oxidise (or the inverse is true in the case of RuHex and TMPD, thus the first step was to oxidise, followed by a reduction) with the results depicted in Figure 4, and their calculated *A_real_* and %*Real* reported in Table 2.

It can be readily observed from the Anson plots that only RuHex goes through the origin (taking into account the double layer capacitance), indicating that *only* this probe is truly outer-sphere, as discussed above (and when considering only the redox probe utilised herein). Again, RuHex, as a near-ideal outer-sphere probe, is the only one that follows Equation (6) for diffusion processes, while the rest need to use the Anson plot for adsorption processes (see Equation (7)) to have a more accurate %*Real*; however, the limitations and incorrect use of this is noted above. Note that deviation from the origin indicates that TMPD and the other redox probes should be classified as inner-sphere redox probes. When comparing the use of CC vs. cyclic voltammetry, given the long list of experimental parameters that need to be taken into account, it is suggested that CC is the most accurate to produce the true electrochemical area and hence %*Real*.

## 4. Conclusions

In summary, for the first time, we have compared methods to determine the real electroactive area of screen-printed electrodes via CV and CC. Details are given to allow novices to utilise these approaches and to understand the various parameters that need to be taken into consideration to determine a robust value for the real electroactive area. Given that Hexaammineruthenium (III) is a near-ideal outer-sphere electron transfer redox probe, it is the optimal choice of analyte to employ when one needs to obtain the most accurate and reliable indication of the real electroactive surface using the Randles–Ševćik equation and the Anson equations/plot. Both of the methodologies estimate similar *A_real_* values using RuHex, with CC being the more accurate. The significance here is not only the utilised methodology itself, but the selection of the redox probe, showing that an ideal outer-sphere probe would be the best to use to obtain the real electroactive area of SPEs and indeed other electrodes. This work is an important and fundamental contribution to those experimentalists who use and benchmark the real electroactive area of screen-printed electrodes since it provides the first comparison of inner- and outer-sphere redox probes, highlighting the various parameters that need to be considered in order to obtain useful estimations of the real electroactive area.

## Figures and Tables

**Table 1:** Calculated electrode areas (*A_real_*) for SPEs using the Randles–Ševćik equations (Equation (2) for quasi-reversible processes of RuHex, TMPD, and capsaicin and Equation (3) for irreversible processes such as NADH, dopamine, and ascorbic acid; note that dopamine has anodic and cathodic processes but is termed irreversible because of the relative rates of electron transfer and mass transport [31]; recorded at 19.6 °C for each redox probe, with the diffusion coefficient used in the calculation. The %*Real*, which is the percentage of *A_real_* divided by the *A_geo_*, is also presented (*N* = 3).

Electroactive Probe	Electrode Area Randles–Ševćik/cm^2^	D/cm^2^ s^−1^	%*Real*
RuHex	0.062	9.10 × 10^−6^	83.25
NADH	0.049	7.40 × 10^−6^	65.47
Dopamine	0.090	6.74 × 10^−6^	120.18
Capsaicin	0.093	7.03 × 10^−6^	123.74
TMPD	0.057	6.32 × 10^−6^	75.64
Ascorbic acid	0.109	1.42 × 10^−6^	145.65

**Table 2:** Calculated electrode areas (*A_real_*) for SPEs using the Anson plot equations (Equations (6) and (7)) recorded at 19.6 °C for each redox probe, with the diffusion coefficient used in the calculation. The %*Real*, which is the percentage of *A_real_* divided by the *A_geo_*, is also presented (*N* = 3).

Electroactive Probe	Electrode Area Anson/cm^2^	D/cm^2^ s^−1^	%*Real*
RuHex	0.055	9.40 × 10^−6^	73.34
NADH	0.077	7.40 × 10^−6^	103.27
Dopamine	0.077	6.74 × 10^−6^	102.51
Capsaicin	0.057	7.03 × 10^−6^	75.91
TMPD	0.053	6.32 × 10^−6^	70.53
Ascorbic acid	0.121	1.42 × 10^−6^	161.21

## Supplementary Materials

The following are available online at http://www.mdpi.com/2079-6374/8/2/53/s1, Figure S1: Topography measurement on a screen-printed electrode (A), height profile (B) from data shown in A and a 3D plot of the topography measurements (C).
