# Supplementary material for: Determination of the Electrochemical Area of Screen-Printed Electrochemical Sensing Platforms
Source: Biosensors (Basel). 2018 Jun 8;8(2):53. doi: 10.3390/bios8020053 (PMC6023085; doi:10.3390/bios8020053)
Supplement: Supplementary file 1 [file biosensors-08-00053-s001.pdf]

Supplementary Material: Determination of the electrochemical area of screen-printed electrochemical sensing platforms

Alejandro García-Miranda Ferrari [1,2], Christopher W. Foster [1,2], Peter J. Kelly [1], Dale A. C. Brownson [1,2] and Craig E. Banks [1,2,*]

[1] Faculty of Science and Engineering, Manchester Metropolitan University, Manchester M1 5GD, UK; alejandro.garcia-miranda-ferrari@stu.mmu.ac.uk (A.G.-M.F.); cwfoster90@gmail.com (C.W.F.); peter.kelly@mmu.ac.uk (P.J.K.); d.brownson@mmu.ac.uk (D.A.C.B.)

[2] Manchester Fuel Cell Innovation Centre, Manchester Metropolitan University, Manchester M1 5GD, UK

* Correspondence: c.banks@mmu.ac.uk; Tel.: +(0)1-612-471-196; Fax: +(0)1-612-476-831

Received: 3 April 2018; Accepted: 6 June 2018; Published: 8 June 2018

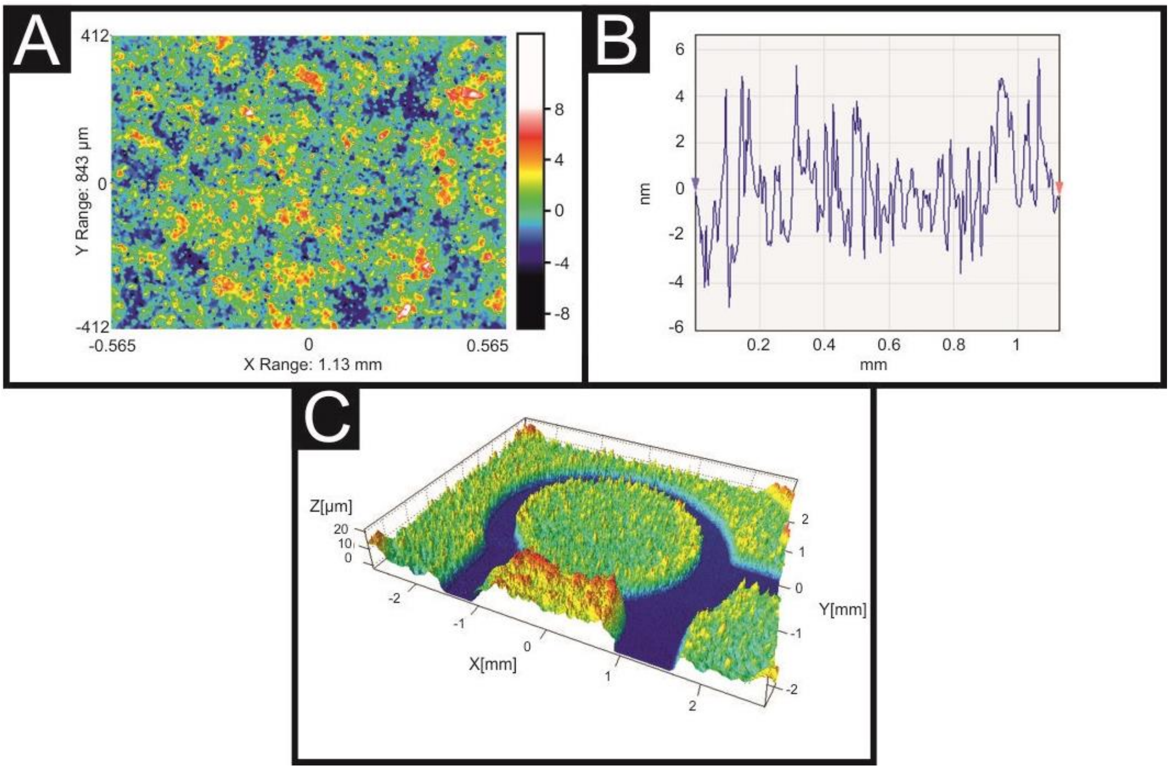

Figure S1. Topography measurement on a screen-printed electrode (**A**), height profile (**B**) from data shown in A and a 3D plot of the topography measurements (**C**).
